# Impact of perineural tumor spread in head and neck adenoid cystic carcinoma for carbon-ion radiotherapy

**DOI:** 10.1016/j.ctro.2025.100928

**Published:** 2025-01-30

**Authors:** Atsushi Musha, Nobuteru Kubo, Hidemasa Kawamura, Naoko Okano, Masahiro Onishi, Takeru Ohtaka, Midori Tamura, Osamu Nikkuni, Yuichi Tomidokoro, Satoshi Yokoo, Kazuaki Chikamatsu, Tatsuya Ohno

**Affiliations:** a Gunma University Heavy Ion Medical Center, Maebashi, Gunma 371-8511, Japan; b Department of Oral and Maxillofacial Surgery and Plastic Surgery, Gunma University Graduate School of Medicine, Maebashi, Gunma 371-8511, Japan; c Department of Otolaryngology-Head and Neck Surgery, Gunma University Graduate School of Medicine, Maebashi, Gunma 371-8511, Japan

**Keywords:** Adenoid cystic carcinoma, Carbon-ion radiotherapy, Perineural tumor spread, Head and neck tumor, Treatment outcome

## Abstract

**Background and purpose:**

Despite recognizing the effectiveness of carbon-ion radiotherapy (C-ion RT) in treating head and neck adenoid cystic carcinoma (HN-ACC), the impact of perineural tumor spread (PNTS) on treatment outcomes and the relationship between PNTS and post-treatment marginal recurrences remain unexplored. Therefore, we analyzed the efficacy of C-ion RT in HN-ACC, focusing on PNTS including details of post-treatment marginal recurrence and indicators for future treatment strategies.

**Materials and methods:**

In this retrospective study, we included 74 patients diagnosed with HN-ACC who underwent C-ion RT between June 2010 and July 2022. Treatment dosages were either 57.6 or 64.0 Gy (relative biological effectiveness) delivered in 16 fractions. Patients undergoing C-ion RT were identified retrospectively from medical records. PNTS was evaluated by magnetic resonance imaging for detecting extratumoral extension to peritumoral nerves.

**Results:**

The median duration of follow-up was 46.4 months. Local recurrence developed in 19 patients. Moreover, most marginal recurrences occurred within the irradiated margins. The 5-year rates for local control (LC), progression-free survival (PFS), and overall survival (OS) were 67.6 %, 47.7 %, and 79.0 %, respectively. LC (*p* = 0.005) and PFS (*p* = 0.013) demonstrated significant variances on analysis based on PNTS occurrence; however, OS did not exhibit a similar pattern.

**Conclusion:**

Our findings highlight the importance for considering disease-specific characteristics and the need for developing targeted strategies that address the complex nature of HN-ACC, particularly in cases involving PNTS, to enhance treatment outcomes and potentially reduce recurrence rates.

## Highlights

• Carbon-ion radiotherapy is safe for head and neck adenoid cystic carcinoma.
• Perineural tumor spread may pose challenges to local control of the tumor.
• Acute adverse events improve with conservative treatment.
• Late adverse events are affected by the tumor-invasion extent of the organ at risk.

## Graphical abstract

**Figure:**
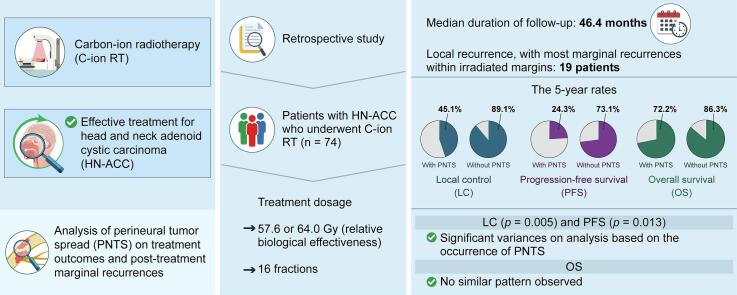

## 1 Introduction

Head and neck adenoid cystic carcinoma (HN-ACC) is a rare cancer, representing approximately 1.8 % of tumors in the head and neck region, as per Japanese statistics [1]. Globally, HN-ACC is infrequent; the standard therapeutic approach entails surgery combined with radiotherapy, yielding favorable outcomes. These include 5-year local control (LC) and overall survival (OS) rates of approximately 77–95 % and 68–85 %, respectively [2], [3], [4]. Nonetheless, extensive resection and subsequent reconstruction pose challenges owing to functional and cosmetic concerns in the head and neck area. In advanced cases, tumors frequently localize adjacent to the orbit or skull base. Hence, carbon-ion radiotherapy (C-ion RT) is garnering attention as a focal treatment modality for managing patients with advanced disease, such as invasion of the orbit or skull base area. A multicenter observational study of C-ion RT in Japan demonstrated a 5-year LC rate of 68 % and a 5-year OS rate of 74 %, showcasing potential outcomes despite most cases being inoperable [5].

Although the efficacy of both surgery and C-ion RT can be assessed, recurrence at the margins or within nerve-infiltrated regions post-treatment poses a challenge owing to the characteristic nerve invasion by ACC [6]. The pathological features of ACC include perineural invasion (PI) and diagnostic imaging or clinical symptoms often reveal perineural tumor spread (PNTS) [7]. Although PI and PNTS are evaluated by different methods, both exhibit characteristics of nerve invasion, implying an association with treatment outcomes, considering the nature of ACC. Following surgery with radiotherapy, ACC recurrence shows histological evidence of PI [8]. Conversely, regarding C-ion RT for ACC, outcomes present histological evidence in the form of solid components [9]. To date, treatment outcomes concerning PNTS, including those of surgery, radiotherapy, and C-ion RT, for ACC have not been elucidated. Therefore, in this study, we evaluated the efficacy of C-ion RT in HN-ACC, with a specific focus on PNTS, including details of post-treatment marginal recurrence and indicators for future treatment strategies.

## 2 Material and methods

### 2.1 Patients

In this retrospective clinical study, we recruited 74 patients, diagnosed with HN-ACC and treated with C-ion RT, between June 2010 and July 2022 at the Gunma University Heavy Ion Medical Center. This study was approved by the Gunma University Review Board (trial approval number: HS2023-044, ethical approval date: July 31, 2023) and conducted in accordance with the Declaration of Helsinki. As this was a retrospective non-invasive study, the description about the study was made available on the Gunma University Review Board website and was approved by the Gunma University Review Board for an opt-out method of consent, by excluding the patients who refused to participate. The patients with ACC who were treated at the Gunma University Heavy Ion Medical Center were included in the multicenter observational study of C-ion RT in Japan [5]. The patients evaluated in the multicenter observational study were treated between November 2003 and December 2014, of which few cases overlap with those in this study.

### 2.2 C-ion RT

We have previously reported the C-ion RT guidelines [10], [11], [12]. The patients were immobilized using thermoplastic shells (Shellfitter; Kuraray, Osaka, Japan) and positioned in customized cradles (Moldcare, Alcare, Tokyo, Japan). In each patient, a customized mouthpiece was secured from the mandible to the maxilla [13]. The XiO-N system (Elekta, Stockholm, Sweden) was used for planning the C-ion RT. Computed tomography (CT; at least 2-mm thickness) was used to plan and delineate the gross tumor volume (GTV) based on contrast-enhanced magnetic resonance imaging (MRI; at least 1-mm thickness). The clinical target volume (CTV) was at least 5-mm margin around the GTV. CTV1 was located around the anatomic sites where the GTV was located, whereas CTV2 was limited to the GTV. The 2-mm margins around CTV1 and CTV2 determine the planning target volume (PTV) 1 and PTV2, respectively. As necessary, these margins were modified when the irradiation areas were in proximity to the organs at risk (such as the eye, optic nerve, brain, or brain stem). The radiation dose was planned for the PTV isocenter. The PTV aimed to adjust the dose to the organs at risk (OAR) with specific considerations and be covered by 95 % of the isodose line of the prescribed dose. The physical dose calculations were referenced using the pencil beam algorithm. The clinical dose distribution was calculated using the physical dose and relative biological effectiveness (RBE) obtained from the responses of the human submandibular gland cells. The dose of C-ion RT was expressed as “Gy (RBE)” [14]. The fraction size was 16; the overall treatment duration was approximately four weeks (four fractions per week). In general, the patients received 64.0 Gy (RBE). In cases of widely irradiated skin and mucosa involvement, we opted for a total dose of 57.6 Gy (RBE). PTV1 was irradiated at 36.0 Gy (RBE) in the first half without specific considerations, and PTV2 in the second half was set at 28.0 or 21.6 Gy (RBE) considering the dose to OAR. Adverse events were evaluated using the Common Terminology Criteria for Adverse Events v5.0.

### 2.3 Patient assessment

These assessments were performed as previously described [10], [12]. Patients who underwent C-ion RT were retrospectively identified based on medical records. We investigated the age, sex, tumor region, TNM stage (Union for International Cancer Control staging criteria, 8th edition), GTV, performance status (PS), and total dose of C-ion RT with or without previous surgery. Recurrence was classified as in-field recurrence for recurrent tumor within the PTV1 field and marginal recurrence for tumors that recurred within and outside the PTV1 evaluated by contrast-enhanced MRI. MRI was performed using a 3-Tesla scanner (MAGNETOM Prisma fit; Siemens Healthcare, Erlangen, Germany) with gadolinium contrast. Images were acquired using a three-dimensional T1-weighted gradient-echo volumetric interpolated breath-hold examination sequence with fat suppression and 1-mm slice thickness. The extent of extratumoral invasion to peritumoral nerves was defined as PNTS [7]. PNTS in this study was diagnosed by a full-time radiation oncologist, who was in charge of C-ion RT in the head and neck region, using this definition. If the contrast-enhanced MRI was indistinct, at least CTV1 was included and treated.

### 2.4 Follow-up

After treatment, the patients were followed up every month for the first year and every 3 months thereafter. CT and MRI were performed every three months, and F-18 fluorodeoxyglucose positron emission tomography (PET) /CT was performed every year. All patients, including those who developed lymph node or distant metastases, were assessed for local effects during the follow-up period until death.

### 2.5 Statistical analysis

Statistical analyses were performed using IBM SPSS Statistics (version 26.0; IBM, Armonk, NY). The LC, OS, and progression-free survival (PFS) rates after C-ion RT were assessed using Kaplan–Meier method and compared using log-rank test. All statistically significant factors from the univariate analysis were fitted in the multivariate analysis using Cox proportional hazards model. Statistical significance was indicated by two-sided p-values < 0.05.

## 3 Results

**Fig. 1:**
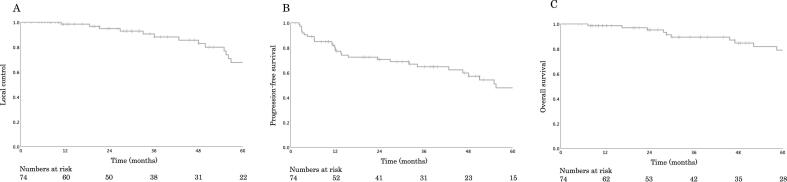
LC, PFS, and OS curves for adenoid cystic carcinoma of the head and neck region treated with carbon-ion radiotherapy. (A) The 5-year LC rate for all patients (n = 74) is 67.6 %. (B) The 3-year PFS rate for all patients is 47.7 %. (C) The 3-year OS rate for all patients is 79.0 %. LC, local control; PFS, progression-free survival; OS, overall survival.

The number of male (n = 36) and female (n = 38) patients was approximately equal (Table 1). The documented primary tumor sites included the paranasal sinus (n = 20), oral cavity (n = 15), nasal cavity (n = 12), parotid gland (n = 9), oropharynx (n = 8), and other sites (n = 10). The majority of the patients was in T4 stage (69 %). The median follow-up duration for all patients was 46.4 (range, 1.2–137.4) months. The patients undergoing C-ion RT as their primary treatment accounted for 89 %, and those with recurrence after undergoing surgery as their primary treatment accounted for 11 %. All patients had gross residual tumor during C-ion RT. The 3- and 5-year LC rates for all patients (n = 74) were 90.5 % and 67.6 %, respectively (95 % confidence interval [CI], 60.9–136.0 %; Fig. 1A). Thirty-six of the 74 patients were below the 95 % PTV dose target due to dose limitation to OAR; 10 of the 36 patients developed local recurrence (mean PTV% was 83.9 %). On the other hand, 26 patients who were below 95 % of the PTV dose target but did not develop local recurrence had a mean PTV% of 76.8 %, a difference that was not statistically significant. The 3- and 5-year PFS rates for all patients (n = 74) were 64.7 % and 47.7 %, respectively (95 % CI, 40.4–70.7 %; Fig. 1B). The 3- and 5-year OS rates for all patients (n = 74) were 89.4 % and 79.0 %, respectively (95 % CI, 80.6–118.4 %; Fig. 1C).

**Table 1:** Patient and tumor characteristics.

Characteristic	
Total (*n*)	74
Age (y), mean (range)	62 (25–85)
Sex, *n* (%)	
Female	38 (51)
Male	36 (49)
Histological type, *n* (%)	
Adenoid cystic carcinoma	74 (100)
Region, *n* (%)	
Paranasal sinus	20 (27)
Oral cavity	15 (20)
Nasal cavity	12 (16)
Parotid gland	9 (12)
Oropharynx	8 (11)
External auditory canal	4 (5)
Nasopharynx	3 (4)
Submandibular gland	2 (3)
Sublingual gland	1 (2)
T stage, *n* (%)	
T1	1 (2)
T2	10 (13)
T3	12 (16)
T4a	22 (30)
T4b	29 (39)
N stage, *n* (%)	
N0	69 (93)
N2b	3 (4)
N2c	2 (3)
M stage, *n* (%)	
M0	60 (81)
M1	14 (19)
GTV (mL), mean (range)	30.3 (3–191)
Perineural tumor spread	
No	28 (38)
Yes	46 (62)
Performance Status (%)	
0	42 (57)
1	30 (40)
2	2 (3)
Total dose (Gy [RBE]), *n* (%)	
57.6	21 (28)
64.0	53 (72)
Previous surgery, *n* (%)	
No	66 (89)
Yes (postoperative recurrence)	8 (11)

Abbreviation: RBE, relative biological effectiveness; GTV, gross tumor volume.

**Fig. 2:**
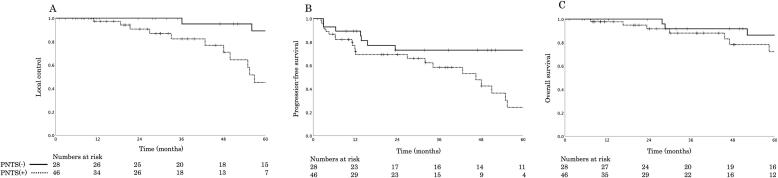
Comparison of PNTS of LC, PFS, and OS curves for adenoid cystic carcinoma of the head and neck region treated with carbon-ion radiotherapy. (A) The 5-year LC rate was 45.1% with PNTS and 89.1% without PNTS. (B) The 5-year PFS rate was 24.3% with PNTS and 73.1% without PNTS. (C) The 5-year OS rate was 72.2% with PNTS and 86.3% without PNTS. PNTS, perineural tumor spread; LC, local control; PFS, progression-free survival; OS, overall survival.

PNTS was reported in 62 % of the patients (Table 1). The 5-year LC rate was 45.1 % with PNTS and 89.1 % without PNTS (Fig. 2A, *p* = 0.005). The 5-year PFS rate was 24.3 % with PNTS and 73.1 % without PNTS (Fig. 2B, *p* = 0.013). The 5-year OS rate was 72.2 % with PNTS and 86.3 % without PNTS (Fig. 2C, *p* = 0.092). In all these comparative analyses, no significant difference was observed between patients with and without PNTS early after C-ion RT such as after 1 or 2 years, with a trend toward differences due to PNTS with longer follow-up, particularly in LC and PFS. LC (*p* = 0.005) and PFS (*p* = 0.013) showed significant differences on comparison based on PNTS using the univariate analysis, whereas OS did not (*p* = 0.092; Table 2). Using multivariate analysis, LC (*p* = 0.003) showed a significant difference, whereas PFS (*p* = 0.11) and OS (*p* = 0.07) did not. No significant differences were observed in univariate and multivariate analyses at a median GTV of 30.3 mL. Moreover, no significant differences in treatment outcomes were found in comparisons based on median age, sex, T stage, PS, total dose, and previous surgery.

**Table 2:** Risk factors for local control, progression-free survival, and overall survival.

		**Local control rate**							**Disease-free survival rate**							**Overall survival rate**						
			Univariate analysis			Multivariate analysis				Univariate analysis			Multivariate analysis				Univariate analysis			Multivariate analysis		
**Factors**	n	5 y rate (%)	HR	95 % CI	*p*	HR	95 % CI	*p*	5 y rate (%)	HR	95 % CI	*p*	HR	95 % CI	*p*	5 y rate (%)	HR	95 % CI	*p*	HR	95 % CI	*p*
**Age**																						
< 61	38	58.3	0.487	0.185–1.287	0.147	0.576	0.44–7.555	0.674	40.8	0.697	0.343–1.414	0.317	0.00	0.00–7.640E + 71	0.92	80.5	2.343	0.885–6.199	0.086	1.432	0.109–18.749	0.784
≥ 62	36	78.1							56.6							77.8						
< 64	40	60.7	0.507	0.179–1.433	0.2	0.514	0.2–13.298	0.688	44.0	0.821	0.405–1.665	0.585	12570.372	0.00–7.556E + 79	0.916	81.9	2.446	0.954–6.268	0.063	3.979	0.285–55.535	0.305
≥ 65	34	76.3							53.6							76.3						
< 69	52	61.9	0.514	0.148–1.785	0.295	1.52	0.168–13.718	0.709	36.9	0.402	0.154–1.046	0.062	0.325	0.096–1.107	0.072	80.0	1.039	0.372–2.902	0.941	0.437	0.108–1.765	0.245
≥ 70	22	82.5							79.2							76.9						
**Sex**																						
Female	38	74.8	0.705	0.280–1.770	0.456	0.251	0.071–0.889	0.032	50.3	0.956	0.472–1.936	0.901	0.861	0.39–1.9	0.711	80.3	0.683	0.275–1.697	0.411	0.494	0.155–1.572	0.232
Male	36	59.6							43.9							78.5						
**T stage**																						
T1–3	23	72.5	1.778	0.687–4.602	0.235	2.045	0.556–7.524	0.282	65.5	2.094	0.933–4.697	0.073	1.522	0.587–3.945	0.388	88.9	1.443	0.551–3.775	0.455	0.846	0.236–3.039	0.798
T4	51	64							39							72.5						
**Perineural tumor spread**																						
No	28	89.1	5.173	1.653–16.192	0.005	8.362	2.049–34.131	0.003	73.1	2.855	1.250–6.520	0.013	2.046	0.851–4.918	0.11	86.3	2.346	0.870–6.329	0.092	2.795	0.92–8.49	0.07
Yes	46	45.1							24.3							72.2						
**GTV (mL)**																						
< 30.3	37	70.6	1.146	0.459–2.860	0.77	0.64	0.17–2.405	0.462	58.8	1.842	0.907–3.737	0.091	1.255	0.542–2.905	0.579	91.7	2.57	0.990–6.668	0.052	3.616	0.907–14.42	0.069
≥ 30.3	37	63.5							36.4							63.3						
**PS**																						
0	42	66.6	0.997	0.403–2.465	0.994	0.813	0.243–2.714	0.736	55.9	1.461	0.723–2.950	0.291	1.317	0.578–2.998	0.512	76.4	0.991	0.400–2.458	0.985	1.792	0.466–6.888	0.396
1, 2	32	68.1							39.8							61.2						
**Total dose (Gy [RBE])**																						
57.6	21	75.5	1.056	0.413–2.700	0.909	0.838	0.236–2.976	0.785	71.5	1.735	0.748–4.025	0.199	1.217	0.495–2.994	0.668	87.2	1.287	0.483–3.431	0.614	0.904	0.281–2.904	0.865
64.0	53	63.6							37.6							74.8						
**Previous surgery**																						
No	66	63.7	0.967	0.220–4.261	0.965	0.741	0.136–4.028	0.728	41.2	0.395	0.094–1.659	0.204	0.477	0.108–2.111	0.329	79.4	0.363	0.048–2.757	0.327	0.822	0.089–7.618	0.863
Yes	8	66.7							66.7							75						

HR, hazard ratio; CI, confidence interval; GTV, gross tumor volume; PS, performance status; RBE, relative biological effectiveness.

Of the 74 patients, 19 showed local recurrence after C-ion RT (Table 3). Median GTV in patients with recurrence was 28.82 (5.15–116.44) mL. Of the 19 patients with recurrence, 12 received a total dose of 64 Gy (RBE) and seven received a total dose of 57.6 Gy (RBE) without significant differences in the total dose (Table 2). Most patients had local recurrence type of marginal recurrence in the irradiated area. The most recurrent areas were the paranasal sinuses in 10 cases; two of the 10 cases showed invasion from this area to the skull base. The shortest and longest local recurrences were in 10.9 and 107.9 months, respectively, and the median was 55.0 months. In 13 of the 19 patients with recurrence and PNTS, maxillary nerve tended to be the most frequently involved area both before C-ion RT and during recurrence. Seven of the 13 patients with PNTS and relapse and half of those without PNTS showed a centripetal (toward brainstem) direction of recurrence.

**Table 3:** Patient characteristics with local recurrence.

No.	Age	Sex	PS	Tumor site	GTV (mL)	T stage	N stage	M stage	Total dose (Gy [RBE])	Perineural tumor spread	Invasion area at C-ion RT	Affected nerve at C-ion RT
1	70	M	0	Oropharynx	7.11	2	0	0	57.6	−	−	−
2	43	F	1	Nasal cavity	17.79	3	0	0	64.0	−	−	−
3	59	F	0	Nasal cavity	16.92	4b	0	0	64.0	+	Pterygopalatine fossa, skull base	Maxillary nerve, facial nerve
4	57	M	0	Paranasal sinus	85.15	4b	0	1	57.6	+	Dorsal maxillary sinus, optic canal	Maxillary nerve, optic nerve
5	63	M	1	Nasal cavity	5.15	1	0	0	57.6	−	−	−
6	48	F	1	Oropharynx	12.58	4a	0	0	57.6	−	−	−
7	59	M	1	Paranasal sinus	77.31	3	0	0	64.0	+	Dorsal maxillary sinus	Maxillary nerve
8	65	F	1	Oral cavity	32.76	4b	0	0	64.0	+	Greater palatine foramen, dorsal maxillary sinus	Greater palatine nerve
9	53	F	0	Oral cavity	27.71	4b	0	0	57.6	+	Lingual tonsil, intrinsic muscles of the tongue	−
10	61	M	1	Paranasal sinus	116.44	4b	0	0	64.0	+	Pterygopalatine fossa, foramen rotundum, inferior orbital fissure	Maxillary nerve, optic nerve
11	60	F	1	Paranasal sinus	41.92	4b	0	0	64.0	+	Dorsal maxillary sinus	Maxillary nerve
12	62	M	1	Oral cavity	25.18	4b	0	1	64.0	+	Dorsal maxillary sinus, greater palatine foramen	Maxillary nerve, greater palatine nerve
13	52	F	1	Parotid gland	18.23	4a	0	0	64.0	+	Pterygopalatine fossa	Maxillary nerve, facial nerve
14	61	F	1	Oral cavity	29.93	3	0	1	57.6	+	Greater palatine foramen	Greater palatine nerve
15	69	M	0	Nasal cavity	23.58	3	0	0	64.0	+	Dorsal maxillary sinus, inferior orbital fissure	Maxillary nerve
16	35	M	0	Oral cavity	9.25	2	0	0	57.6	−	−	−
17	70	F	0	Oropharynx	51.94	4b	2c	0	64.0	−	−	−
18	54	M	0	Paranasal sinus	42.5	4a	0	1	64.0	+	Dorsal maxillary sinus	Maxillary nerve
19	70	M	0	Paranasal sinus	54.14	4a	0	0	64.0	+	Pterygopalatine fossa, infratemporal fossa	Maxillary nerve

No.	Local recurrence type	Local recurrence area	Affected nerve at recurrence	Direction of recurrence	Local recurrence period	Distant metastasis area	Distant metastasis period	Salvage treatment	Follow-up period after recurrence	After treatment survival
1	In-field	Tongue base	−	In field	107.3	−	−	C-ion RT	30.1	alive
2	Marginal	Paranasal sinus	−	Centripetally	86.6	−	−	BSC	48.5	alive
3	Marginal	Nasal cavity − Skull base	Maxillary nerve	Centripetally	48.1	Brain	48.1	BSC	21.0	alive
4	Marginal	Paranasal sinus	Maxillary nerve	Centripetally	33.1	Lung	14.0	BSC	14.0	dead
5	Marginal	Nasopharynx	Maxillary nerve	Centripetally	107.9	−	−	Cyberknife	11.0	dead
6	Marginal	Tongue base	−	Centrifugally	72.3	Lung	72.3	Cyberknife	39.3	alive
7	Marginal	Paranasal sinus	Maxillary nerve	Centrifugally	42.8	−	−	Cyberknife	18.9	alive
8	Marginal	Paranasal sinus	−	Centrifugally	55.5	Lymph node	55.5	BSC	2.9	dead
9	Marginal	Tongue base	−	Centrifugally	61.0	Lung	61.0	BSC	14.6	alive
10	Marginal	Paranasal sinus	Maxillary nerve	Centripetally	26.9	−	−	Surgery	17.1	alive
11	Marginal	Paranasal sinus	−	Centrifugally	98.5	−	−	BSC	3.1	alive
12	Marginal	Paranasal sinus	Maxillary nerve	Centripetally	18.6	Lung	11.2	BSC	4.8	dead
13	Marginal	Skull base	Maxillary nerve	Centripetally	55.0	−	−	C-ion RT	41.0	alive
14	Marginal	Paranasal sinus	−	Centrifugally	56.9	Lung, bone	6.3	Palliative radiotherapy	7.2	dead
15	Marginal	Orbital apex	Mandibular nerve, maxillary nerve	Centripetally	49.9	−	−	BSC	16.6	dead
16	Marginal	Optic canal	Maxillary nerve	Centripetally	56.2	−	−	C-ion RT	11.0	alive
17	Marginal	Oropharynx	−	Centrifugally	36.2	−	−	TS-ONE ®	13.8	alive
18	Marginal	Paranasal sinus − Skull base	Optic nerve, maxillary nerve	Centripetally	10.9	−	−	C-ion RT	13.3	alive
19	Marginal	Paranasal sinus − Skull base	−	Centrifugally	21.3	Bone	21.3	C-ion RT (Local), Cyberknife (bone)	16.5	alive

PS, performance status; GTV, gross tumor volume; RBE, relative biological effectiveness; C-ion RT, carbon-ion radiotherapy; BSC, best supportive care; TS-ONE ®is a fluoropyrimidine anticancer drug.

Acute grade-2 mucositis and grade-1 dermatitis were the most common acute adverse events (Table 4). In all cases, acute adverse events improved immediately after conservative therapy. However, some late adverse events such as conjunctivitis, trismus, oral fistula, and osteoradionecrosis did not improve immediately and required long-term symptomatic treatment. Patients who developed oral fistulas and osteoradionecrosis showed prominent bone invasion by tumor. Patients who developed trismus showed significant tumor invasion of the masticatory muscles. Ophthalmologic disorders, such as cataracts, optic nerve disorder, and intraocular hemorrhage, were a result of prominent intraorbital invasion and a highly advanced tumor, necessitating the need for high dose.

**Table 4:** Acute and late adverse events in all patients receiving carbon-ion radiotherapy (n = 74).

Acute		Any grade	Grade 1	Grade 2	Grade 3	Grade 4
	Mucositis	46	6	31	9	−
	Dermatitis	58	48	8	2	−
	Xerostomia	5	5	−	−	−
	Dysgeusia	9	7	2	−	−
	Conjunctivitis	4	2	2	−	−
Late						
	Mucositis	20	6	12	2	−
	Dermatitis	57	47	8	2	−
	Xerostomia	9	7	2	−	−
	Dysgeusia	7	4	3	−	−
	Conjunctivitis	20	6	12	2	−
	Trismus	20	17	3	−	−
	Oral fistula	8	1	6	1	−
	Osteoradionecrosis	23	1	15	7	−
	Brain necrosis	6	2	3	−	1
	Brainstem necrosis	2	2	−	−	−
	Nasal congestion	8	8	−	−	−
	Chronic sinusitis	4	3	1	−	−
	Olfactory nerve disorder	2	2	−	−	−
	Middle ear infection	8	3	5	−	−
	External otitis	−	−	−	−	−
	Intraocular hemorrhage	6	1	1	1	3
	Cataract	1	−	−	1	−
	Optic nerve disorder	1	−	−	−	1
	Nasolacrimal duct obstruction	12	11	1	−	−

## 4 Discussion

In this study, the proportion of patients with T4 disease was 69 %, 5-year LC rate was 67.6 %, and OS was 79 %. These results are comparable to previously reported results of C-ion RT for HN-ACC by the National Institute for Quantum Science and Technology [9], in which 54 % had T4 with a 5-year LC rate of 68.6 % and OS of 74.8 %. In addition, although dose dependence of treatment outcomes has been reported [9], this study did not show dose dependence. In the multicenter observational study that reported dose dependence of the salivary gland cancer on C-ion RT, the rate of ACC was 48 % and the biologically effective dose of Japanese original RBE model implicated in LC was 89.6 Gy (RBE) [15]. Similarly, an ACC study of 113 cases in a retrospective study on C-ion RT in the National Institute for Quantum Science and Technology confirmed a dose-dependent advantage in LC [9]. On the other hand, a multicenter observational study of C-ion RT in Japan on oral non-squamous cell carcinoma, including 47 % ACC [16], confirmed no-dose dependence in LC. In the present study, no dose-dependent effect on treatment outcome in 74 cases of ACC was identified. Studies in which dose dependence was confirmed tended to have a larger proportion of oral cases than did the present study and others in which dose dependence was not confirmed, suggesting a possible effect of primary minor salivary glands, which are more common in the oral cavity. On the other hand, in radiotherapy, major salivary gland cancer has been reported with a radiation dose ≤ 66 Gy and was a significant dependent predictor of good LC, but the rate of ACC was low at 18 % [4].

Surgery and postoperative radiotherapy are recommended for HN-ACC in operable cases [2], [3], [4]; the results of surgery and postoperative radiotherapy are better than those of C-ion RT, including 5-year LC rate of 77–95 % and 5-year OS rate of 68–85 % [2], [3], [4]. However, these reports mostly describe T stages T1–3 with approximately 20–40 % of T4 cases. Although the T4 rate is much higher than that of surgery and radiotherapy, C-ion RT has a favorable outcome. Therefore, C-ion RT is a viable treatment strategy for HN-ACC, particularly in advanced cases. Based on previous clinical trials, C-ion RT is likely to respond favorably to ACC. For an accurate comparison with surgery, a clinical trial in early T1–3 cases or a propensity match study in early cases should be considered. However, the collection of patient data is difficult, specifically since surgery is still considered the first choice for early-stage cases.

Most local recurrence types were marginal in this study. The median recurrence duration was 55.0 (range: 10.9–107.9) months. Therefore, patients with ACC after C-ion RT should continue to be followed up with imaging studies, even in cases wherein LC is good for over 5 years. This may be a reason for marginal recurrence due to the lack of C-ion doses in the marginal invasion area or the areas of lesion invasion not detectable on imaging. Because of the large number of T4 cases in this study, many patients had extensive tumor invasion and PTV < 95 % due to dose constraints of OAR. However, no significant difference was observed between insufficient PTV-prescription dose and marginal recurrence, and the marginal recurrence of ACC in C-ion RT may not be due to insufficient dose alone. The percentage of PI in ACC is relatively high, such as 66.7 % [17], 59 % [4], and 58.6 % [8]. The presence of PI plays a role in the recurrence rates [8]. Owing to the small sample volume of biopsies in most cases in this study, the occurrence of nerve invasion was not confirmed in most cases. Future studies are warranted to determine the impact of C-ion RT and PI on treatment outcomes. Regarding PNTS, 62 % of cases were confirmed. The LC and PFS were statistically significant in the PNTS group with a particular advantage in the LC group. These results indicated the importance of the C-ion dose at the margins of tumor invasion. On the other hand, PNTS occurrence seems to be associated with LC, specifically marginal recurrence. Therefore, we suggest that in cases of imaging features such as PNTS, a wider irradiation field is necessary when considering treatment planning, rather than uniformly determining the irradiation field because invasion into areas that cannot be confirmed on imaging is strongly suspected. Based on the characteristics of the recurrence direction in this study, considering expanding the extent of CTV1 in the centripetal direction in head and neck ACC would be appropriate in general, regardless of PNTS. Particularly, there is a particular case of primary oral (soft and hard palate) lesion without PNTS to discuss about centripetal direction of invasion (Table 3, case 16). A pathway of spread invasion primarily starting from the palate in centripetal direction to the greater palatine nerve, pterygopalatine fossa, maxillary nerve, trigeminal ganglion is considered. Subsequently, tumor is thought to have destroyed the surrounding tissues and invaded the optic canal and orbit. However, according to this study, since PNTS positivity is associated with local recurrence, this consideration should be emphasized more in cases of PNTS. Although PET has been used and evaluated for PNTS in recent years, MRI is currently the gold standard [18]. In addition to MRI data, when ACC infiltrates a nerve, clinical signs of nerve involvement should be reflected in the treatment plan, since clinical signs of nerve involvement develop in the area of the innervating nerve and symptoms are detected on medical examination, which is an important factor in demarcating the radiation area, particularly at the margins [7]. In contrast, in cases of comprehensive CTV, less than 60 Gy in patients undergoing postoperative radiotherapy and less than 70 Gy in those receiving definitive radiotherapy are associated with recurrence; accurately identifying the margin to be irradiated and prescribing the dose are important [19].

The adverse-event onset was similar to that reported in previous C-ion RT studies [5], [9], [10], [11], [12], [13] with acute adverse events improving with conservative treatment and late adverse events influenced by the extent of tumor invasion to the OAR. Therefore, the adverse events observed in this study, reported in many patients with T4, included oral fistula, osteoradionecrosis, brain necrosis, intraocular hemorrhage, and optic nerve disorder. Therefore, obtaining informed consent prior to treatment initiation, not only for treatment efficacy, but also for the possible acute and late adverse events in every patient is necessary.

This study has a few limitations. This study included a small number of cases at a single institution. Prospective clinical studies need to be conducted at multiple centers in the future, but the approach regarding marginal recurrence needs to be considered. This is a primary consideration for expansion of the irradiation field to reduce the rate of marginal recurrence. However, this should be carefully considered because enlarging the irradiation field may increase the incidence of adverse events while improving LC. Prospective multicenter clinical studies should be conducted in the future, specifically to improve LC rates and dose-prescription ranges. Since CTV1 is based on the first half of the 36-Gy prescription, expanding this region has a low risk of increasing adverse events in terms of dose limitation [20]; however, the effect of the tumor control by expanding the low-dose region is unknown. Regarding head and neck ACC, we recommend expanding the range of CTV1 in the centripetal direction regardless of PNTS. However, if patient has PNTS, we would consider expanding CTV1 in a more centripetal direction. The rate of local recurrence may be reduced by expanding the irradiation edge in consideration of the anatomical structure of the patients with PNTS as a characteristic feature.

## 5 Conclusions

Although this study confirmed the efficacy and safety of C-ion RT in HN-ACC, disease-specific clinical features, such as PNTS, may challenge LC. These insights also highlight the importance for future research to refine C-ion RT strategy, including dose optimization and better treatment area delineation, to improve care in patients with HN-ACC.

## CRediT authorship contribution statement

**Atsushi Musha:** Conceptualization, Data curation, Formal analysis, Funding acquisition, Investigation, Methodology, Project administration, Resources, Software, Validation, Visualization, Writing – original draft, Writing – review & editing. **Nobuteru Kubo:** Investigation, Methodology, Project administration, Resources, Visualization, Writing – review & editing. **Hidemasa Kawamura:** Investigation, Methodology, Project administration, Resources, Visualization, Writing – review & editing. **Naoko Okano:** Investigation, Methodology, Project administration, Resources, Visualization, Writing – review & editing. **Masahiro Onishi:** Investigation, Resources, Writing – review & editing. **Takeru Ohtaka:** Investigation, Resources, Writing – review & editing. **Midori Tamura:** Investigation, Resources, Writing – review & editing. **Osamu Nikkuni:** Supervision, Writing – review & editing. **Yuichi Tomidokoro:** Supervision, Writing – review & editing. **Satoshi Yokoo:** Supervision, Writing – review & editing. **Kazuaki Chikamatsu:** Supervision, Writing – review & editing. **Tatsuya Ohno:** Supervision, Writing – review & editing.

## Declaration of Competing Interest

The authors declare that they have no known competing financial interests or personal relationships that could have appeared to influence the work reported in this paper.
